# Formation and Discharge of Zn Sponge Anodes, Followed by Synchrotron Hard X‑ray Imaging

**DOI:** 10.1021/acsaem.5c01110

**Published:** 2025-07-14

**Authors:** Benedetto Bozzini, Nicola Sodini, Alexander P. Kao, Alessio Veneziano, Lucia Mancini

**Affiliations:** † Department of Energy, 18981 Politecnico di Milano, v. Lambruschini 4, 20156 Milano, Italy; ‡ 18474 Elettra - Sincrotrone Trieste S.C.p.A., S.S. 14 - km 163.5 in Area Science Park, 34149 Basovizza (Trieste), Italy; § Departament d’Enginyeria Mecànica, 16777 Universitat Rovira i Virgili, Avinguda Països Catalans núm. 26, 43007 Tarragona, Spain; ∥ Department of Materials, Slovenian National Building and Civil Engineering Institute (ZAG), Dimičeva ulica 12, 1000 Ljubljana, Slovenia

**Keywords:** Zinc anode, zinc battery, zinc sponge, X-ray microtomography, synchrotron radiation, *in situ*, *in operando*, formation

## Abstract

The fabrication of engineered Zn anodes often relies on different forms of ZnO as the material in direct contact with the alkaline aqueous electrolyte in the pristine assembled cell state. Of course, in this case, the as-assembled cell is in the discharged state and requires an initial charging step, or “formation”, to generate active metallic Zn. The formation of ZnO-based anodes is a complex process the control of which calls for an in-depth understanding of electrochemical phase growth. In fact, formation gives rise to morphochemical imprinting, profoundly impacting the electrode functional performance. The present work contributes to the understanding of the formation of Zn sponge electrodes, combining electrochemistry and synchrotron-based X-ray imaging. Specifically, we employed dynamic *in operando* radiography to select the potentiostatic formation conditions that exclude hydrogen-induced damaging of the sponge structure. Subsequently, formation and the subsequent first discharge are followed by time-lapse *in situ* tomography, allowing to track the early structural evolution of the sponge electrode and the Zn/ZnO phase distribution.

## Graphical abstract

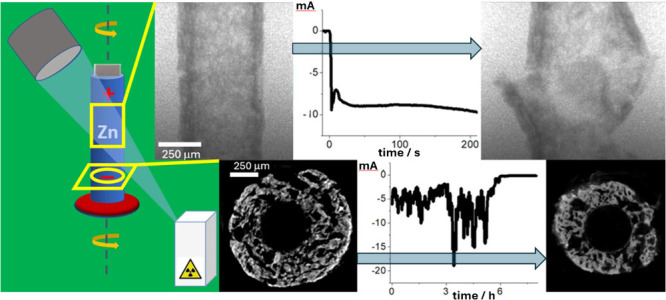

## 1 Introduction

Worldwide, the increasing need of reliable and sustainable energy supply, storage and portability, combined with global industrial competition, imposes a stringent schedule for battery research and development. Specifically, next-generation rechargeable batteries for electric vehicles and energy storage applications, bear promise of an effective solution for hydrocarbon-free electric mobility and balancing of intermittent renewable sources. Among the different technologies being considered, rechargeable zinc-air batteries (RZABs) are promising candidates owing to their comparatively high specific energy, moderate cost, abundant and distributed raw-material resources, environmental friendless and safety.^1,2^ Although primary Zn-air batteries can be regarded as a well-assessed technology, at least for low-power applications, real-life implementation of RZABs is hindered by a series of poorly understood fundamental issues. This situation is mainly due to a lack of solid understanding of the role played by the microstructure of the different battery components and of its evolution during operation. In view of technologically acceptable RZAB cyclability, the single most critical component is surely the Zn anode. In fact, it is has been clear for decades that the irreversibility of microstructural modifications of the anode is one of the key processes leading to degradation and failure. Nevertheless, Zn shape change mechanisms have been investigated in a way that has not led to conclusive knowledge, so far. In particular, on the one hand, the existing data—in most cases referring either to the anode operation on an integral, whole-electrode scale, or addressing electrode structure *ex situ*—is not adequate to support clear guidelines for anode improvement. On the other hand, the available mathematical and physicochemical models are generally descriptive rather than predictive. The lack of quantitatively reliable materials-science parameter values is certainly one of the reasons why mathematical modeling has in practice not been as effective as hoped. To counteract cycling instabilities of the Zn anode, several solutions—sometimes based on questionable physicochemical principles—have been published,^3–9^ of course, accounting for the relevant literature in an exhaustive way is beyond the scope of this paper.

Six classes of approaches can be recognized: (i) use of a range of additives (organic, polymeric and inorganic) in the classical alkaline aqueous electrolytes; (ii) selection of alternative electrolytes (weakly acidic aqueous, organic, ionic liquids); (iii) implementation of different types of separators; (iv) modification of anode configuration in the battery layout; (v) surface modification of the Zn anode and (vi) tailoring of the anode microstructure (sponges, flakes and fibers). Among microstructure tailoring schemes, a particularly promising method is the fabrication of Zn sponge electrodes, consisting in a network of connected metallic Zn branches, covered with a layer of ZnO (for more details, see Section 2.1).^10–14^ In the operation of these materials, on the one hand the continuity of the electron-conductive metallic zinc network is presupposed to persists down to the required depth of discharge (DOD) as well as over cycling and, on the other hand, Zn­(II) is expected to be confined into the porous framework of the electrode. If there requirements be met, in principle, extensive cycling without formation of dendrites and loose Zn or ZnO particles—that have become inactive owing to detachment from the external electronic circuit—would be possible. Positive experimental evidence on the actual volumetric distribution of Zn and ZnO *in operando*—in combination with integral electrochemical information at the cell scale and performance figures of merit—is, of course, crucial for the understanding of the actual behavior of these materials and for their knowledge-based improvement. In this framework, the relevance of developing better capabilities of directly imaging morphological and chemical changes, as they occur within the operating battery, is evident.

In the recent past, imaging studies on RZAB anodes have mainly concentrated on *ex situ* analyses of dendrite formation. However, more recent advances in imaging methods and electrochemical cell design have enabled *in situ* investigations e.g. of Li, Pb and Zn dendrites, based on nuclear magnetic resonance, confocal laser scanning microscopy, FIB-SEM as well as transmission X-ray microscopy.^15–19^ X-ray imaging techniques, X-ray and neutron tomography in particular, presently allow the direct *in situ* contextual imaging and characterization of complex microstructures on length scales from millimeters down to tens of nanometers. Laboratory- and synchrotron-based X-ray computed microtomography (SR-mCT) has been successfully employed to investigate the performance of Zn anodes, as well as air cathodes, in operating zinc-air batteries at the micron scale.^20–25^ In fact, in addition to a scanning electron microscopy analysis, the use of X-ray imaging allows to adopt a nondestructive method for a morphotextural sample characterization on millimeter to centimeter-sized samples. The acquisition of data allows to visualize and segment features such as pores and cracks, as well as crystalline and amorphous phases within an electrode element or in the assembled cell. Then, parameters such as electrode porosity and connectivity, distribution (number and sizes) of particles and cracks, tortuosity—controlling effective transport properties—can be evaluated. Aging and failure analyses, based on microstructural data, can thus be performed and fed into appropriate mathematical models.

In a paper of ours^14^ we have investigated for the first time Zn sponge electrodes with a multitechnique approach, centered on laboratory-based X-ray mCT, combining electrochemical and morphological observations. This study was centered on the long-term evolution of the anode material, for which the space-time resolution enabled by a laboratory X-ray source was appropriate. In particular, the mCT results of ref 14 allowed both to deepen the understanding of the multistep fabrication protocol and to assess the structural effects of anode cycling. The purpose of the present paper is instead to focus on the dynamics of the formation process, leading to Zn metal formation and on the initial stages of Zn reoxidation. material evolution. To this aim, a high space- and time-resolution is required, that mandates access to SR-mCT. In fact, synchrotron-radiation imaging measurements offer high-spatial and contrast resolution capabilities, allowing to extract accurate morphotextural parameters describing the electrode microstructures on a sufficiently large, and technically representative, probed volume.

## 2 Experimental Section

### 2.1 Fabrication of Zn Sponge Anodes

The Zn sponge fabrication strategy is to prepare an anode in the discharged state, consisting of a highly porous, branched network of Zn/ZnO structures with electronically percolating Zn cores carrying a ZnO outer layer. This approach has been pioneered in ref 10, developed in further work by the same group^26–28^ and continued also by other workers.^29–31^ This configuration, on the one hand, ensures efficient ionic and electronic contact to ZnO via electrolyte from the pore side and through the Zn framework on the core side, and, on the other hand, minimizes the risk of building up dead Zn or ZnO fragments, as a result of battery cycling. During the first charge, or formation, the initial ZnO layer, in contact with the electrolyte, is converted to metallic Zn. During subsequent discharge, by selecting appropriate depth of discharge (DOD) levels, a fraction of the external structure is converted back to ZnO, without oxidizing the core and thus allowing the persistence of a highly percolating metallic skeleton. During the following charge step, depending on the depth of charge (DOC) a selected amount of ZnO is converted back to Zn. Moreover, during discharge, zincates are released to the electrolyte and the porous structure of the sponge electrode is in principle capable of immobilizing them. In this way the electroactive species are confined spatially close to the electrode. Thus, suppression of zincate diffusion away from the electrode/electrolyte interface, in turn can suppress the concentration gradients that favor shape changes and dendrite formation during recharge (see e.g. ref 32 and references therein). In this way, significant durability improvement can be achieved, with respect to Zn anodes exhibiting different active-material arrangements.^33–37^

Zn sponge anodes, exhibiting the X-ray absorption properties appropriate for X-ray imaging at mean X-ray energies down to ca. 27 keV, were fabricated after a protocol inspired by ref 10. Specifically, process improvements were implemented in ref 14, in view of precise electrode shaping. In the present study, we further fine-tuned the Zn sponge fabrication protocol, to improve the anodic functional properties and to cope with the optical requirements of SR-mCT. Briefly, anode fabrication is carried out in three steps: (i) formulation of a spreadable Zn-paste; (ii) anode shaping and (iii) heat-treatment. The preparation of a spreadable precursor paste instead of the castable slurry proposed in ref 10, allows the production of thin green electrodes, directly pasted onto the current collector. This spreadable paste was obtained by blending: 6 g of micron-sized Zn powder (Todini and Co S.p.a., Monza, Italy); 7 g of sodium dodecyl sulfate (Panreac Applichem); 0.21 g of carboxymethylcellulose (CTS); 1 mL of water and 2.3 mL of *n*-pentane (Merck). The mixing process has been improved with respect to ref 14, by employing an IKA RW20 mechanical stirrer. Euristically, better paste homogeneity was found to improve electrode quality in the case of thinner layers. Subsequently, the paste was generally rolled to a thickness of ca. 400 μm onto graphite rods, 500 μm in diameter, with a homemade plane-parallel glass rolling device that enabled relatively accurate thickness control for the achievement of optimal X-ray attenuation. For fast time-resolved experiments, in which a lower radiation absorption would favor contrast, we also used 100 μm thick electrodes. As far as X-ray imaging requirements are concerned, the choice of graphite current-collectors is motivated by the fact that is material is both highly electronically conductive and transparent in the X-ray energy range of interest in this work. Choice of metallic current collectors would either totally hinder X-ray transmission or critically impact the contrast required to image and segment a porous Zn/ZnO electrode. Graphite is slight suboptimal from the strictly electrochemical viewpoint, as detailed below, but this suboptimality is by far compensated by the fact that it enables X-ray imaging. The rod length was 30 mm and the coating was applied for 20 mm from the rod tip. The nominal active surface area of the electrode was ca. 0.82 cm^2^. As hinted at above, graphite enhances to some extent Zn self-discharge, but, in any case, all types of current collector materials for particulate or porous Zn anodes, in which the contact between the electrolyte and the current collector cannot be excluded by construction, exhibit some degree of galvanic coupling. In fact, the current-collector material is invariably noble than Zn, thus contributing to self-discharge.^38^ Since currently, the material of choice for Zn-anode current collectors is Cu.^39^ while Ni is also sometimes considered in the literature,^40^ we have tested the behavior of graphite against these two metals. To this aim, we have selected zero-resistance ammetry (ZRA), since this electrochemical methods yields direct information about the short-circuit current and the mixed potential vs Hg/HgO of a galvanic couple. ZRA measurements, carried out in the electrolyte used for tests, aerated 6 M KOH solution, showed that graphite gives rise to a degree of galvanic coupling that is intermediate between those of Cu and Ni (Figure 1) and can thus be regarded as an acceptable solution, in view of its crucial advantages for X-ray imaging, highlighted above. In fact, Cu exhibits the best behavior, with a mixed potential that is very close to the open-circuit potential (OCP) of Zn and a vanishing short-circuit current density. Ni, instead, yields a notable galvanic coupling, owing to its high catalytic activity toward the hydrogen evolution reaction (HER). Graphite exhibits an intermediate behavior, that is pretty acceptable for the imaging experiments at stake in the present research. As in ref 14, the green anodes are first sintered in Ar at 400 °C for 2 h and then calcinated in air at 650 °C for 2 h. The lower-temperature stage in Ar eliminates the organic fraction, creating a porous structure, and leads to the formation of a reticulated Zn structure held together by necking between Zn particles. The higher-temperature step in air consolidates the Zn network and generates a ZnO shell around it.

**Figure 1:**
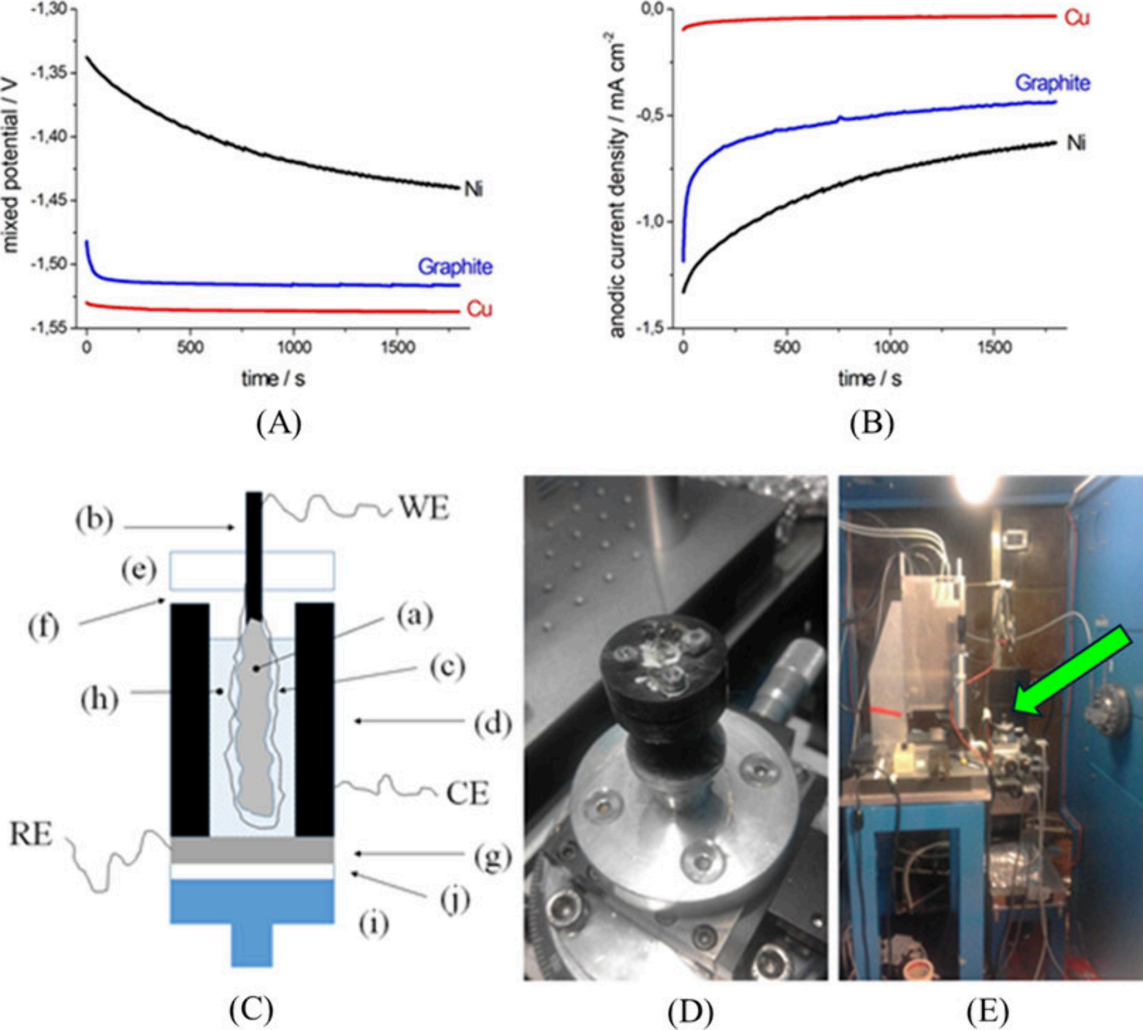
(A, B) Zero-resistance ammetry data (A: mixed potential vs Hg/HgO; B: anodic current density) for galvanic coupling of Zn in aerated 6 M KOH aqueous solution to candidate current-collector materials: Cu, graphite and Ni (surface area ratio: Zn/current-collector = 2). Cu exhibits the best behavior and Ni the worst one (see main text for details). (C) Scheme of the cell for *in situ* and *in operando* SR-mCT (not to scale). (a) Zn sponge anode; (b) graphite rod (anode support); (c) separator; (d) graphite cylinder (cell body and air cathode); (e) insulating cap to fix anode and connect it to the cell body; (f) ports for air access; (g) Zn disk (cell bottom and reference electrode); (h) electrolyte; (i) stab for mounting on rotator; (j) insulating layer. WE, CE, RE: electrical connections to working, counter and reference electrodes, respectively. (D) The cell, mounted on the rotating sample stage. (E) Same, in the context of the tomography cabinet: the position of the cell is indicated with a green arrow.

### 2.2 Electrochemical Measurements

Electrochemical measurements—consisting in OCP, potentiostatic charge and discharge and galvanostatic discharge experiments—were performed with a VersaSTAT 3 potentiostat. The purpose of electrochemical measurements in this work is impose formation and first discharge conditions for *in situ* imaging conditions. The only exception, is a set of measurements aimed at selecting the best type of current-collector for the imaging experiments at stake in this research. Related electroanalytical work and more extended electrochemical measurements targeting material preparation and long-term evolution, have been the object of a companion paper^14^ and will not be replicated here.

All electrochemical experiments were carried out with the graphite-supported Zn sponge anodes prepared as detailed in Section 2.1, and mounted in the cell for synchrotron imaging, described in Section 2.4. Measurements were performed in a three-electrode configuration with a Zn quasi-reference electrode (RE), mounted as detailed in Section 2.4. The electrolyte was an aerated 6 M KOH aqueous solution.

### 2.3 Electrochemical Cell for X-ray Imaging Experiments

The Zn-air cell developed for *in situ* SR-mCT is shown in Figure 1C–E. For the present investigation, we developed a dedicated cell. In fact, the cell configurations described in the scanty literature on X-ray imaging of Zn anodes are appropriate for solid Zn electrodes, but cannot be employed for sponge electrodes. The key cell components are sketched in Panel (A). The Zn sponge anode ((a): working electrode, WE) was fabricated as described in Section 2.1. Electrical contact to the active layer was obtained by connecting the uncoated part (b) of the graphite rod with the external circuitry. The anode is wrapped with a layer of Celgard 3501 (c), separating the anode from the free electrolyte. The cathode is a graphite cylinder ((d): counter electrode, CE) which also plays the role of the cell body and the X-ray window. In this way, the whole electrochemically active region of the anode can be imaged. The anode is fixed to the cell body with an insulating cap (e), that is sealed with an alkali-resistant glue (f) to the graphite cell body.

The graphite cylinder is fixed, again with an alkali-resistant glue, to a Zn disk (g) which acts as the bottom of the cell and the quasi-RE. The cell is filled with electrolyte (h) and fixed to a stab for mounting on the rotator (i) through an insulating layer (j). Electrical connections to the three electrodes are ensured by Kapton-coated Cu wire, 0.2 mm in diameter, secured mechanically with a system of nuts and bolts and fixed by Ag paste (Panel (B)). The insulated wires are long and flexible enough to accommodate a suitably large number of cell rotations (Panel (C)).

### 2.4 X-ray Imaging Experiment and Data Treatment

The experiments were performed at the SYRMEP beamline at the Elettra synchrotron laboratory in Basovizza (Trieste, Italy). It is worth noting that synchrotron mCT, compared with mCT based on laboratory sources, benefits of the following key characteristics: (i) an X-ray flux that is several orders of magnitude higher, also enabling high temporal resolution; (ii) large energy range, with possibility to monochromatize the beam; (iii) parallel beam, yielding detector-limited spatial resolution; (iv) high spatial coherence, that enhances phase-based contrast modes. Details of the SYRMEP facilities and available instrumentations have been described in references 41 and 42 ; synchrotron imaging experiments were performed using a combination of dynamic (*in operando*) microradiography (mXR) and mCT measurements. The schematic layout is given in Figure 1C while a photo of the setup is given in Panels (D) and (E) of Figure 1. At SYRMEP the bending magnet source delivers a polychromatic nearly parallel laminar-shaped X-ray beam with a maximum area of 100 mm (horizontal) × 6 mm (vertical) at 15 m from the source. In our case, we operated using a filtered beam (filters: 1.5 mm Si plus 1.0 mm Al) corresponding to a mean X-ray energy of 27 keV. For the acquisition of both mXR sequences and mCT scans, a macroscope camera equipped with an air-cooled, 16-bit, 2048 × 2048 pixels, CCD detector (Photonic Science, UK) was employed. The detector optical system is based on the indirect detection of X-rays: a 25 μm thick single-crystal LuAG:Ce scintillator screen, used to convert X-rays into visible light was lens-coupled to the CCD camera. In order to work in propagation-based phase-contrast mode,^43,44^ the sample-to-detector distance was set to 200 mm.

#### 2.4.1 *In Situ* Time-Lapse Computed Microtomography

Part of the samples were imaged through *in situ* time-lapse SR-mCT measurements, varying the charge/discharge cycling conditions. For each sample submitted to tomographic scanning, a set of 1500 radiographic images (or projections) was acquired with the detector at equiangular steps over a full rotation angle of 180°, setting an isotropic pixel size of 1.07 μm. The exposure time/projection was varied between 2 and 4 s, depending on the sample transmission. Thus, the total time for a scan varied between 1 and 2 h, including software overheads.

The tomographic reconstruction was performed using the custom-developed SYRMEP_Tomo_Project (STP) software suite.^45^ The same software was used for phase retrieval applied to projections (prior to reconstruction)—using the single-distance Paganin algorithm^46^—and for ring artifact removal. We reconstructed the data using two different values, namely 5 and 50, of the gamma ratio (i.e., the ratio between the real and imaginary parts of the refraction index of the material) during phase retrieval. A value of 5 was selected to optimize the visibility of pores, keeping a high spatial resolution (negligible blurring in the image). A value of 50, instead, allowed to increase the contrast between the Zn and ZnO phases, despite of a limited blurring of the images.

The 32-bit float reconstructed images have been converted to 16-bit TIFF format for 2*D*/3D visualization and further data processing.

#### 2.4.2 Dynamic *in Operando* Radiography Conditions

A second group of samples was investigated by dynamic *in operando* mXR. The experiments were carried out acquiring sequences (with variable number of images, depending on the electrochemical conditions) of sample radiographs at constant exposure time. The output data consist of a time series of 2D mXR images. In order to map the dynamic anode behavior under electrochemical polarization, sequences of radiographs were collected, acquiring an image every 4 s. For corrections, 5 images of dark current, 5 flat field beam images, and a radiograph of the pristine sample were taken before starting the electrochemical experiment.

#### 2.4.3 Image Processing, Analysis and Visualization

The SR-mCT images of the graphite-supported Zn sponge anodes were processed and analyzed by using the *Pore3D* software library, custom-developed at the Elettra facility.^47^ Prior to reconstruction, noise was removed from the projections applying a 3D median filter. Then, the reconstructed 16 bit raw volumes were masked to select only the region of the images occupied by the sample, in view of quantitative image analysis, thus excluding the background. A two-dimensional (2D) masking was applied to individual images, using the following procedure. (i) Application of an automatic Otsu thresholding provided the first estimation of the position and shape of the region of interest in the images. (ii) Subsequent iterations of dilation and erosion processing operators filled the whole region of the Zn sponge anodes. (iii) Finally, a single numerical value was assigned to the identified background, in order to separate it from the region of interest.

In order to differentiate between voids and solid phases (ZnO and Zn), a segmentation procedure of the masked volumes of interest has been carried out through an automatic 3D Multi-Otsu algorithm,^48–51^ assigning different phases to different classes of gray level. The algorithm was set to 3 classes and results are binary volumes composed of porous, Zn and ZnO phases. Pores and metallic Zn represented by less than 8 voxels in the volume were neglected in the subsequent quantitative analyses.

The porosity of Zn anodes and the distribution of metallic Zn resulting from the three investigated conditions (pristine, as formed and after the first discharge) have been then analyzed. The quantity and distribution of the porous and solid phases in correspondence of each of the electrode conditions were assessed using a 3D skeletonization analysis based on the LKC method,^52^ as implemented in the *Pore3D* software. This operation reduces the morphology of a 3D digital object to a network of nodes and branches that preserves the geometric and morphological features of the original object. We used the LKC skeleton algorithm and the computed parameters within the 3D structure to quantify the connectivity of the porous phase and of metallic Zn. In particular, to assess the connectivity of the metallic Zn phase, we used the Backbone connectivity approach.^53^ In this context, the backbone of a 3D structure is defined as the set of the object portions, the elements of which are all connected to each other, keeping each individual backbone separated from the other ones. The number of backbones within a 3D structure is used to quantify the interconnectivity of the material under analysis: in the case of the sponge Zn anodes of interest, the higher the number of backbones, the less interconnected is the overall metallic framework. Specifically, the connected components of the skeleton were calculated, to identify each isolated backbone. The connectivity parameter was then calculated as the ratio between the volume of the largest backbone and the total volume of the metallic Zn phase. Moreover, to capture the morphological variations resulting from the application of the electrochemical polarization, we also estimated the fraction and size of pores: both open and closed ones. The fractional porosity—a quantity ranging from zero to one—was evaluated as the proportion between the volume occupied by the segmented pores and the total volume of the anode.

The pore size was extracted using the Skeleton Analysis module of *Pore3D*. First, the 3D volume of segmented pores underwent skeletonization; then, we applied the Maximal Spheres method^42^ to the skeletonized structure. In the Maximal Spheres method, the diameter of the maximal sphere enclosed within a pore is calculated. Finally, the obtained pore size distribution was fitted with a log-normal distribution, whence mean pore sizes and their standard deviations were estimated. It is worth noting that all computations related to the porous phase were performed using data reconstructed with a phase retrieval gamma ratio of 5, while the ones related to the ZnO and Zn phases were done using data sets reconstructed with a gamma ratio of 50. This approach has been used in order to emphasize spatial or contrast resolution and to ease the segmentation of the phases of interest.

The visualization of raw and processed 2D images, both for tomographic sections and mXR sequences, has been obtained by using the open source *Fiji* software.^54^ The workflow employed for the analysis of SR-mCT images and selected VOIs of the anodes is summarized in Figure 2.

**Figure 2:**
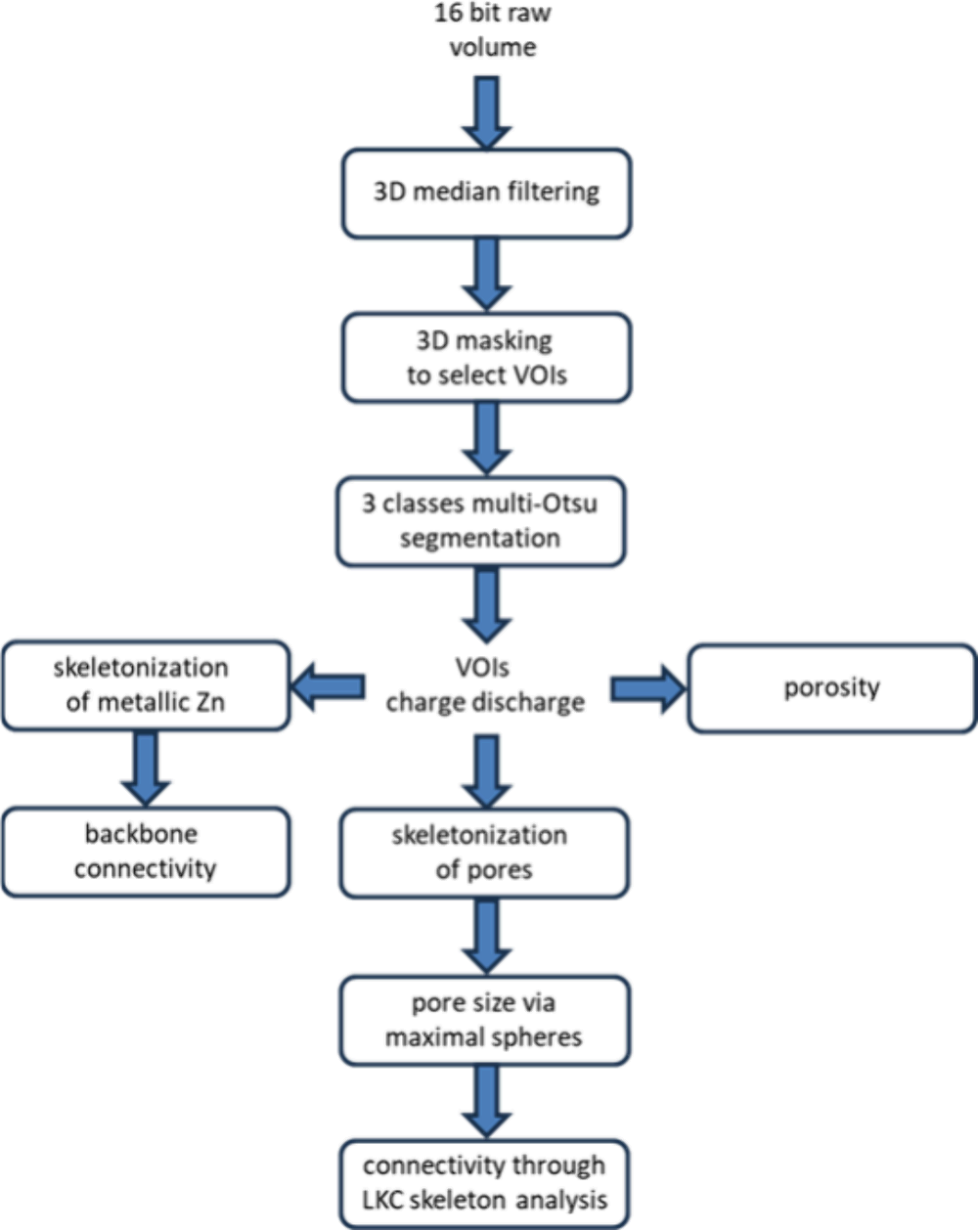
Schematic of the workflow employed for the analysis of SR-mCT images and selected volumes of interest (VOIs).

The Authors would be very pleased to share the raw scan data with interested Colleagues. Please, contact the Corresponding Author to agree the data transfer mode.

## 3 Results and Discussion

### 3.1 Formation of as-Fabricated Zn Sponge Anodes: *in Operando* Radiography

A crucial point in Zn anode formation—i.e. the first charging process for anodes fabricated in the discharged state, as mentioned in Section 2.1, eq 1—in an aqueous electrolyte, is the concurrent HER (eq 2). In fact, this process, in addition to lowering the Coulombic efficiency, because the total current *i*_tot_ is the sum of a Zn-reduction current *i*_Zn_ and an HER current *i*_HER_ (eq 3), can lead to mechanical damaging of the electrode microstructure. Since H_2_ gas bubbles evolve precisely at the current-collector/electrolyte interface, they cause a pressure between the current-collector and the Zn/ZnO composite, that can bring about active material detachment.^55,56^ In this context, graphite, though not electrocatalytic for HER, nevertheless, exhibits a higher activity than Zn, that in fact is an HER poison. In addition, for the present purposes, ZnO can be regarded as HER inactive.

1 $$ZnO+H_{2}O+2e^{-}\to Zn+2OH^{-}$$

2 $$2H_{2}O+2e^{-}\overset{at\;graphite\;surface}{\to }H_{2}+2OH^{-}$$

3 $$i_{tot}=i_{Zn}+i_{HER}$$

Of course, the formation process is controlled by electrochemical means that—with the exception of weakly acidic electrolytes^57^—can hardly be used to diagnose HER onset. Thus, a knowledge-based approach requires dedicated monitoring of gas evolution and mechanical integrity. The only method for HER monitoring reported in the literature so far, to the best of the Authors’ knowledge, is mass spectrometry recently proposed in ref 58. To this aim, in this work. we followed for the first time the evolution of anode morphology by *in operando* radiography under potentiostatic formation conditions: the chronoamperometric curves are reported in Figure 3A and the sequences of radiographs are depicted in Panels (C)–(E) of Figure 3. As in ref 14, the current density refers to the nominal anode area. It can be noticed (Figure 3C and D) that the application of −150 and −100 mV vs Zn leads to a high HER rate. This, in turn, triggers the destruction of the Zn sponge, that can be followed by dynamic radiography and monitored with the drop in cell current (black and red plots of Figure 3A). This current drop is due to the loss of reducible ZnO and the high HER overvoltage at the residual graphite rod. Electrode destruction is chiefly caused by the formation of H_2_ gas bubbles at the current-collector/electrolyte interfaces within the porous structure, for the electrocatalytic reasons mentioned in the previous paragraph. Instead, polarizing the electrode at −50 mV vs Zn, the sequence of radiographs (Figure 3E) shows a stable structure. Here only a fragment is fluctuating as a result of limited HER rate. In correspondence, the cell current initially exhibits an increase, due to the extension of the active area as a result of progressive Zn reduction (Figure 3A, blue plot, corresponding to a DOC of ca. 16%).

**Figure 3:**
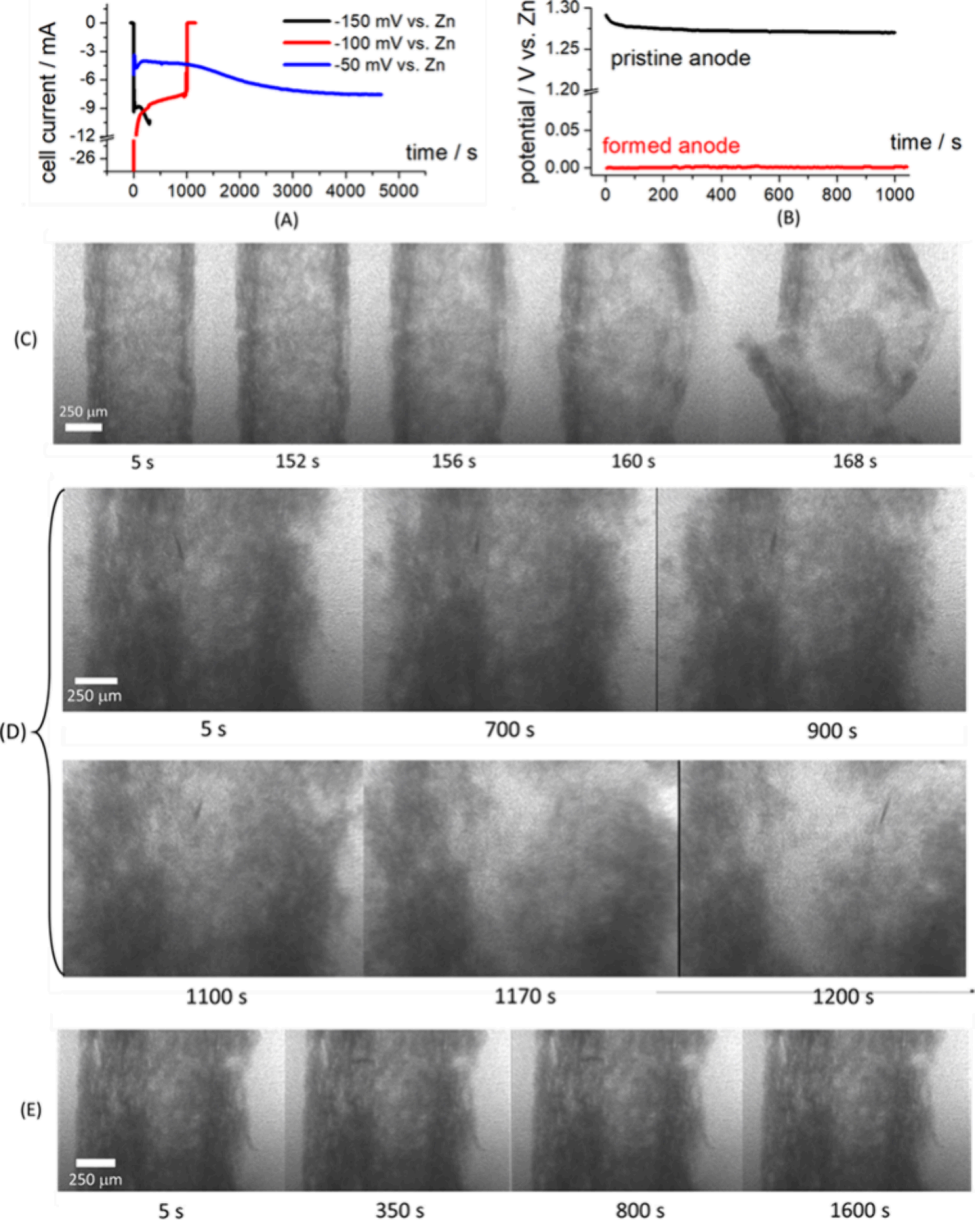
(A, B) Electrochemical measurements for the cell with sponge electrode. (A) Chronoamperometries during formation (charging) at the indicated potentials, corresponding to *in operando* radiography measurements of Panels (C)–(E). The experiment carried out at −150 mV vs Zn is started with applied potential, but without the electrolyte, that is added at time 0 s. (B) Open-circuit potential (OCP) of the anode in pristine, uncharged (black plot) and formed (charged, red plot) at −50 mV (conditions of blue plot of Panel (A)). (C–E) *In operando* X-ray radiographies of pristine Zn sponge electrode, polarized at: (C) −150 mV vs Zn (black plot of Panel (A)); (D) −100 mV vs Zn (red plot of Panel (A)); (E) −50 mV vs Zn (blue plot of Panel (A)).

The OCP values of the cell in the pristine state and after the potentiostatic formation step at −50 mV are reported in Figure 3B. The OCP value of the cell in the pristine, discharged state (black plot) is typical of a mixed-potential condition, the numerical value of which is dominated by the graphite substrate, in contact with the electrolyte through the porosities of the spongy structure.

After formation (charging, red plot), the potential is essentially that of metallic Zn. The electrode, the formation of which was followed by radiography (Figure 3E), has been further analyzed, in order to monitor the 3D structural evolution corresponding to quantitative reduction. To this aim, after having dismounted the cell from the sample stage and having held it at OCP for ca. 1 h, we polarized it cathodically again at −50 mV vs Zn for ca. 8 h (Figure 4C).

**Figure 4:**
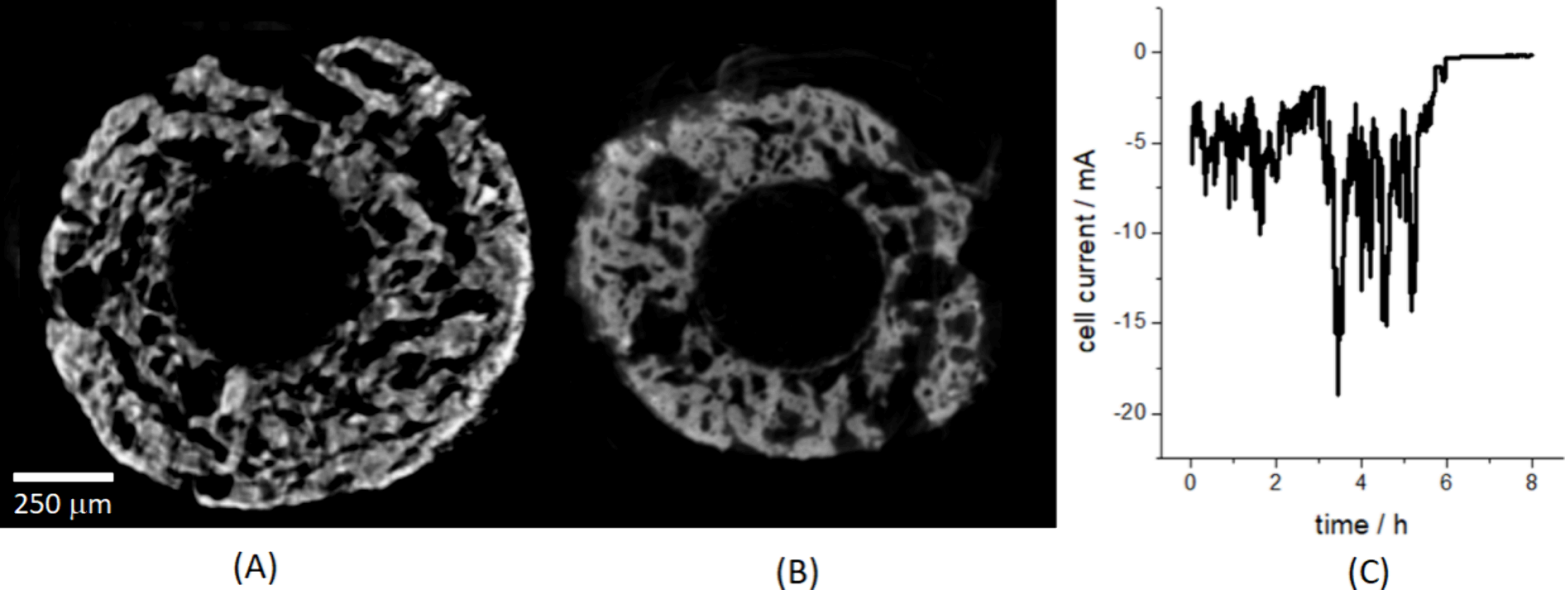
(A) *In situ* tomographic axial slice of the cell used for *in operando* radiography, after prolonged application of cathodic polarization of −50 mV vs Zn (Figure 3E). (B) For comparison, representative *in situ* tomographic axial slice of a pristine cell at OCP. (C) Cell current time series resulting from the application of −50 mV vs Zn after formation (see details in text).

The electrochemical response was characterized by current relaxation toward zero in ca. 1 h, denoting essentially total conversion of ZnO to Zn. Of course, since no Zn^2+^ was present in the pristine electrolyte (see Section 2.2) no net plating of Zn can occur, but just conversion of ZnO to Zn, either directly through solid-state reactions or indirectly through a solution-mediated process. In a recent publication, we proved that the latter mechanism prevails with ZnO-based composites in aqueous electrolytes.^59^ This explains the profound morphological modifications accompanying full reduction of the electrode and leading to an increase of the apparent volume of the object (see Panels (A) and (B) of Figure 4). The high noise level in this measurement might be due either to higher electrical disturbancies outside the hutch or to the detachment of Zn fragments from the electrode, leading to partial, temporary short circuits between the WE and the CE.^60–62^ After having mounted the cell back on the rotator, a tomographic scan was recorded at the end of the extensive reduction process: a representative axial slice is reported in Figure 4A).

Unfortunately, due to lack of time, we decided not to record a tomographic scan of the initial state of the same electrode, but we compared it with a typical axial slice of a pristine sample in the electrolyte at OCP (Figure 4B). Evident metal crystallization features appear, accompanied by the closure of the smaller open pores and enlargement of the larger ones.

### 3.2 Charge and Discharge of Zn Sponge Anodes, *in Situ* Microtomography

The formation process brings about irreversible transformations of the electrode material, resulting in a characteristic imprinting, that drives subsequent electrochemical processes and influences cycle life. Of course, a systematic investigation of these aspects would be a long-term research project, but in the framework of the present investigation, we endeavored to follow the electrode material evolution during the first discharge step following formation. Charge and discharge of a Zn sponge anode were thus investigated by *in situ* SR-mCT in the following representative conditions: (i) as fabricated; (ii) after formation by potentiostatic charging and (iii) after galvanostatic discharge. As discussed in Section 3.1, potentiostatic formation allows better chemical control, while galvanostatic discharge is the most diagnostic testing method (for details, see, e.g. ref 63 and references therein). As detailed in Section 2.4.3, the tomographic scans were segmented for pores and the ZnO and Zn phases. The corresponding electrochemical tracks are reported in Figure 5. 3D imaging experiments of anode structure evolution upon formation and first discharge were started recording a tomographic scan at OCP of the pristine anode as mounted in the cell in contact with the electrolyte (electrochemical trace in Figure 5A, black plot). As expected, this voltage trace is very similar to that recorded with the cell employed for *in operando* radiography.

**Figure 5:**
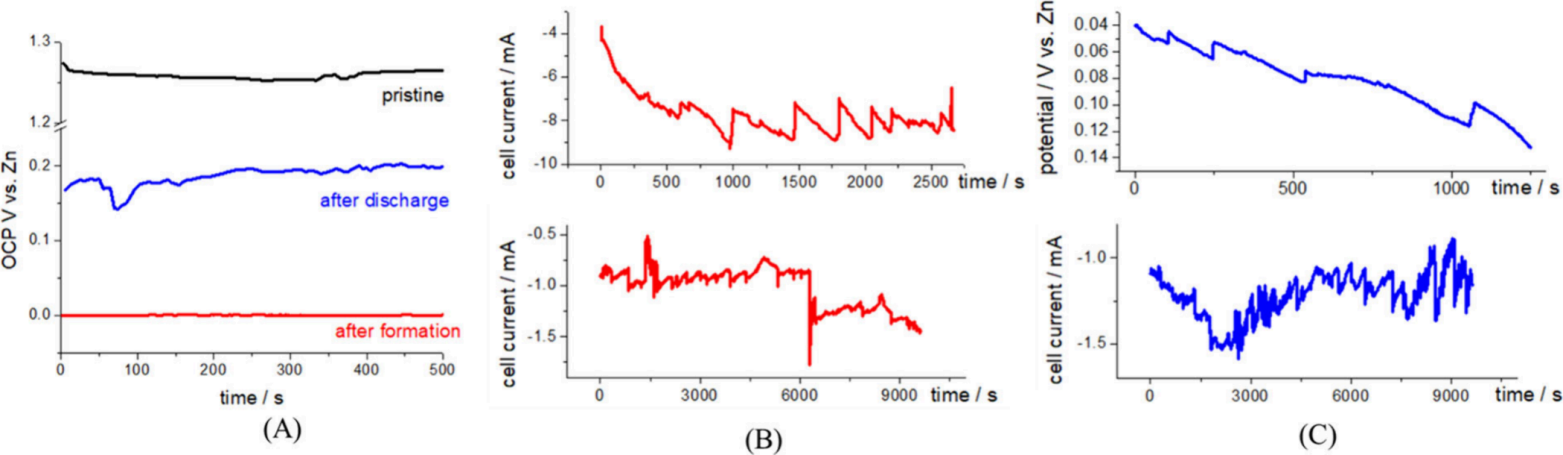
Electrochemical measurements corresponding to the *in situ* tomography scans of Figure 6. (A) Open-circuit potential (OCP) of the anode for the following conditions. Black plot: pristine conditions; red plot: as-formed (immediately after the charge of Panel (B), upper panel); blue plot: immediately after the discharge of Panel (C), bottom panel. (B) Chronoamperometries: upper panel, during charging at −50 mV vs Zn; bottom panel, holding period at the protection potential of 0 mV vs Zn during tomographic scanning. (C) Upper panel: chronovoltammetry during discharge at cell current 10 mA; bottom panel: chronoamperometry during the holding period at the protection potential of 0.2 V vs Zn during tomography.

A typical axial slice for the pristine electrode at OCP is shown in Figure 6A. The raw data have been segmented for pores (Figure 6D), Zn (Figure 6G) and ZnO (Figure 6J), showing the three key components of the electrode.

**Figure 6:**
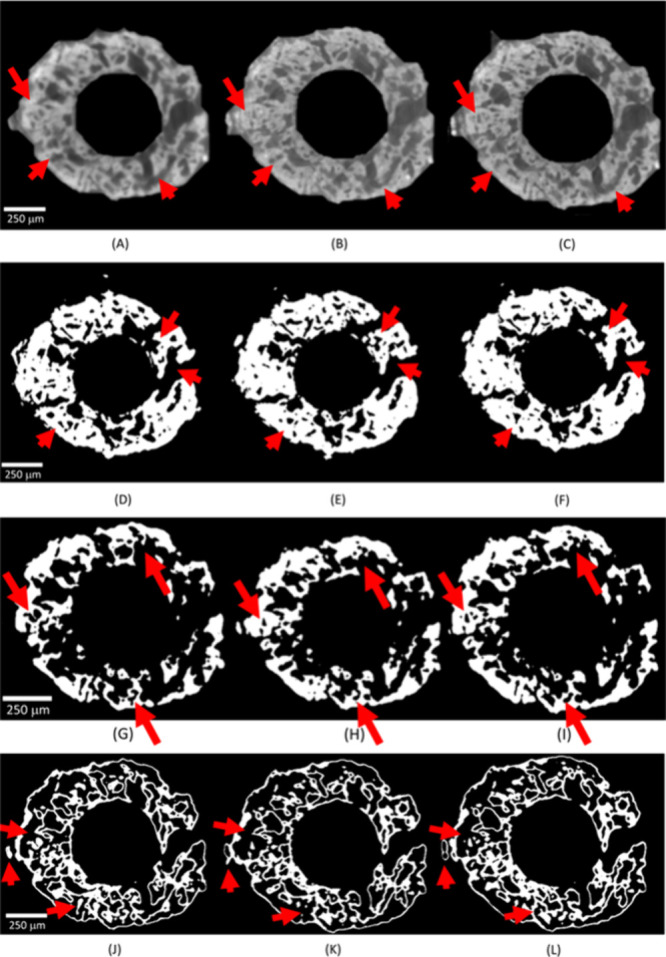
(A–C) A typical *in operando* tomographic axial slice of the Zn sponge anode under the following conditions: (A) pristine electrode at open-circuit potential (OCP); (B) after the formation period (see Figure 5B); (C) after the discharge period (see Figure 5C). (D–F) Images corresponding to Panels (A)–(C) segmented for pores. (D) Pristine electrode; (E) after formation; (F) after discharge. (G–I) Images corresponding to Panels (A)–(C) segmented for Zn. (G) Pristine electrode; (H) after formation; (I) after discharge. (J–L) Images corresponding to Panels (A)–(C) segmented for ZnO. (J) Pristine electrode; (K) after formation; (L) after discharge.

It is worth noting that, thanks to the phase-contrast modality, Zn and ZnO can be clearly distinguished, showing the typical features of the as-fabricated material with a Zn branched core coated with a ZnO shell.

To follow the morphochemical evolution of the anode resulting from the formation process, we adopted this strategy: (i) we first charged the anode potentiostatically at −50 mV vs Zn for 45 min, corresponding to a depth of charge (DOC) of ca. 11% (Figure 5B, upper plot). (ii) After formation, the measured the OCP value (Figure 5A, red plot) was, as expected, very close to 0 mV vs Zn. (iii) In order to compensate for self-discharge, we imposed a potentiostatic polarization of 0 mV vs Zn during the tomography scan (Figure 5B, bottom plot). The chronoamperometry during formation (Panel (B), upper plot) is very similar to that recorded at −50 mV vs Zn during the dynamic radiography test (Figure 3A, blue plot).

The tomographic axial slice for the charged electrode, corresponding to the pristine one of Figure 6A, is reported in Figure 6B and the segmentations for pores, Zn and ZnO in Panels (B) of Figure 6D–L.

Evident changes in the morphologies of the individual phases, some of which are highlighted with red arrows, can be noticed: enlargement of the open pores and formation of new pores due to the transformation of ZnO to Zn (eq 1), with a lower specific volume, increase of the dimensions of the Zn zones and contraction of the ZnO regions. Quantitative evaluations extracted from the segmentation are reported in Figure 7 and will be discussed below.

**Figure 7:**
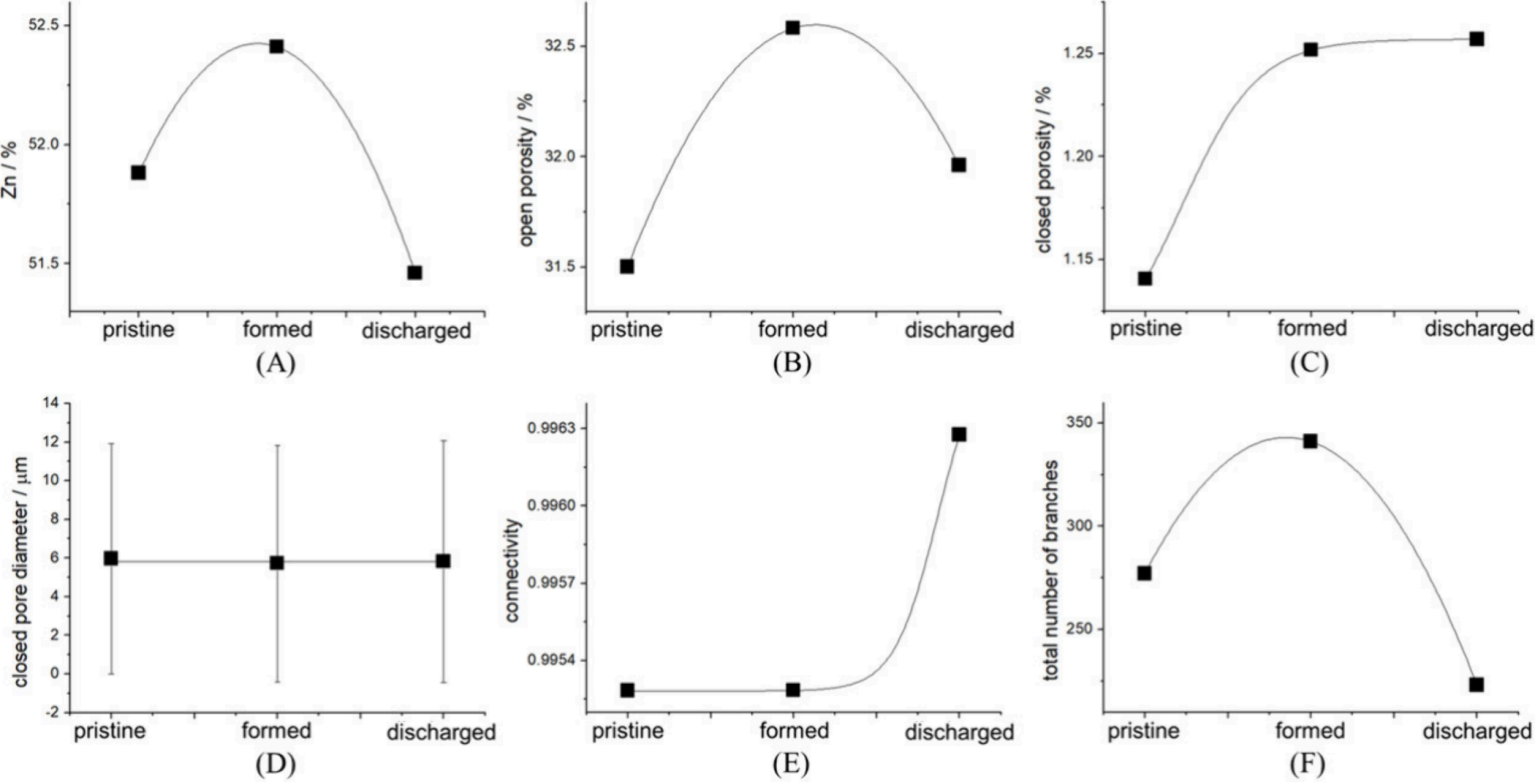
Quantitative analyses of the anode volume segmentations of *in operando* tomographic scans (Figure 6) as a function of electrochemical conditions (see Figure 5). (A) Volume fraction of Zn; (B) open porosity; (C) closed porosity; (D) closed pore diameter; (E) connectivity and (F) total number of Zn branches. The continuous lines are guides for the eye.

After formation, the anode was discharged galvanostatically at 10 mA for 1250 s to a depth of discharge (DOD) of ca. 7% (Figure 5C, upper plot). The discharge curve is typical of a conversion electrode, exhibiting an essentially flat voltage profile. In addition, the slight overvoltage serrations can be explained with the transient formation of oxide layers that—thanks to Zn structuring—are not capable of achieving the critical thickness of ∼2 μm, causing stable passivation.^58,64^

Finally, after discharge, the OCP value was recorded (Figure 5A, blue plot) showing values that denote the establishment of a mixed potential, though with still a strong contribution from metallic Zn. In order to counteract self-discharge during the tomographic scan, we followed the same strategy adopted after charge: we applied potentiostatic conditions corresponding to the OCP value of 0.2 V recorded after the galvanostatic discharge step (Figure 5C, bottom plot). The tomographic axial slice for the discharged electrode is shown in Figure 6C, corresponding to the same position of the electrode examined in pristine and as-formed conditions (Panels (A) and (B)). The segmentations for pores, Zn and ZnO corresponding to the raw image of Figure 6C are reported in Figure 6F, I and L. Again, clear morphological changes of the individual phases can be assessed, now corresponding to Zn transformation to ZnO: contraction of the open pores due to ZnO growth from Zn, decrease of the dimensions of the Zn zones and expansion of the ZnO regions.

In Figure 7, we present the results of quantitative analyses, described in Section 2.4.3 and Figure 2, of the segmented anode volumes in the three investigated conditions. We found that, among the sponge anode architecture morphology estimators evaluated, only three are sensitive to the electrochemical operating conditions investigated in this work: (A) volume fraction of Zn; (B) porosity and (C) connectivity. The Zn volume fraction changes upon formation and discharge in a way that reflects the DOC and DOD. In fact, the formation process implies the conversion of ZnO to Zn, according to reaction eq 1, while during the subsequent discharge, Zn is converted back to ZnO, following the same reaction running in the opposite direction. The possibility of directly monitoring the buildup and space-time distribution of metallic Zn during the formation process provides handles for a systematic screening of the operating conditions for this crucial fabrication step. Moreover, tracking the conversion of the Zn framework to ZnO provides the knowledge basis to maximize the anode capacity without damaging the electronically percolating structure of the sponge electrode.

It is worth noting that the Zn fraction of the as-prepared Zn sponge is smaller with the improved fabrication protocol adopted for this work, in comparison with that employed in ref 14. In fact, the better dispersion of Zn particles leads to a higher degree of oxidation of the sponge in the last step of the heat-treatment (see Section 2.1). Panel (B) shows that open porosity increases during formation and decreases during the subsequent discharge step, as a result of the conversion of ZnO to Zn with attending volume contraction of the compact part of the electrode architecture and of the growth of lower-density ZnO crystallites during discharge. Thanks to the improved fabrication approach, closed porosity (Panel (C)) is very low and exhibits only a slight increase with the formation/first discharge sequence, owing to material rearrangement in anodic and cathodic phase growth processes. Of course, keeping closed porosity low is an advantage because it decreases electrode toughness and volumetric density, without contributing to triple-phase boundary density. The limited variation of closed porosity is a result of the electrochemical inactivity of these regions. Instead, upon prolonged cycling, as pinpointed in ref 14, pore closure can occur as a result of reciprocated plating-stripping. In keeping with very small variations of closed porosity, closed pore diameter (Panel (D)) does not change measurably.

In line with our approach of ref 14, we assessed the consistency of the Zn framework, that is expected to be a crucial factor for functional performance. To this aim, we estimated the degree of connectivity of the Zn phase (Figure 7E). As detailed in Section 2.4, after having evaluated the lengths of the connected Zn branches, the connectivity is computed as the ratio of the longest branch of the network to the total length of the Zn network (*backbone* approach). In Panel (F) we report the total number of branches. It can be noticed that formation and discharge to DOC/DOD of ca. 10% does not appreciably affect the connectivity of the Zn framework. Moreover, the value of the connectivity parameter of the anodes produced with the improved protocol is considerably higher with respect to the values found in ref 14. Instead, the total number of branches of the Zn phase was found to follow the electrochemical processes occurring during charge and discharge: it increases with cathodic Zn formation and it decreases upon oxidation of Zn to ZnO.

## 4 Conclusions

This work addresses the chemical, structural and morphological aspects of the phase-growth processes accompanying the formation and subsequent first discharge of Zn sponge electrodes. Since the last step of Zn sponge electrode fabrication is an oxidation one, it leads to the growth of a continuous ZnO layer, coating the entire metallic skeleton. Thus, notwithstanding the presence of a percolating metallic Zn framework that imparts mechanical consistency, electronic conductivity and part of the capacity of the anode, the material in contact with the electrolyte—generally an alkaline aqueous solution—is ZnO. An initial charging step, denominated “formation” in the battery literature, is therefore required to activate the anode, transforming an appropriate proportion of ZnO into Zn. Of course, this process implies electrochemical phase formation and potentially leads to modifications of the sponge structure, affecting its porosity and connectivity. Evidently, subsequent discharge leads to the reformation of ZnO: a process that, in turn, impacts the geometrical and structural characteristics of the metallic sponge. All these phenomena, and the corresponding consequences on the anode cycling performance, critically depend on electrochemical operating conditions. Electrochemical measurements and *post mortem* structural analyses are highly informative, but *in situ*, chemically sensitive imaging work provides better handles to understand these processes. A pioneering work of ours^14^ has opened up the use of X-ray microcomputed tomography (mCT)—based on laboratory sources—for study on this class of electrodes. The present work further develops this approach, presenting the first synchrotron-based *in operando* microradiography (mXR) and *in situ* mCT study of Zn sponge anodes. The higher dynamic and space resolution capabilities enabled by the synchrotron source allowed to address the formation step and initial stages of discharge.

The selection of formation conditions is known to be critical, because Zn shape change and concurrent HER can impair the sponge structure. In this work, we applied a range of potentostatic conditions and followed dynamically the sponge structure by dynamic *in operando* mXR. We thus directly imaged in time, HER-induced structural damaging, brought about by the application of excessively cathodic conditions, allowing a knowledge-based selection the optimal formation potential.

Subsequently, the morphochemical electrode modifications, corresponding to formation at the optimal potential and the first discharge, were followed by time-lapse *in situ* mCT. The growth of the Zn phase during formation and that of ZnO during discharge could be tracked, together with the evolution of open and closed porosity. Analyses of 3D reconstructions allowed to quantify the evolution of electrode porosity, Zn framework connectivity and relative amounts of Zn and ZnO.

In addition to specific electrochemical materials science results, the present work pinpointed the technical requirements and highlighted the capabilities of SR-mCT, opening up this approach for subsequent work that will explore systematically the fabrication, formation and cycling parameter space for Zn sponge anodes, resorting to complementary synchrotron- and lab-source mCT exploiting their respective characteristics. Moreover, the approach presented in this work would enable direct access to morphological information regarding electrochemical phase formation processes in a broad range of applications including, among others: evolution of battery conversion anodes and cathode, metal and polymer electroplating, semiconductor fabrication as well as corrosion science and technology. This would provide unique handles to correlate the details of solid material modifications with the electrolyte chemistry and electrochemical conditions.
